# Probing the Gold/Water Interface with Surface-Specific Spectroscopy

**DOI:** 10.1021/acsphyschemau.2c00044

**Published:** 2023-01-04

**Authors:** Stefan M. Piontek, Dennis Naujoks, Tadneem Tabassum, Mark J. DelloStritto, Maximilian Jaugstetter, Pouya Hosseini, Manuel Corva, Alfred Ludwig, Kristina Tschulik, Michael L. Klein, Poul B. Petersen

**Affiliations:** † Faculty of Chemistry and Biochemistry, Ruhr-Universität Bochum, 44801 Bochum, Germany; ‡ Faculty of Mechanical Engineering, Institute for Materials and ZGH, Ruhr-Universität Bochum, 44801 Bochum, Germany; § Institute for Computational Molecular Science, Temple University, Philadelphia, 19122 Pennsylvania, United States; ∥ Max-Planck-Institut für Eisenforschung GmbH, 40237 Düsseldorf, Germany; ⊥ Light Conversion Inc., Vilnius City Municipality, Vilnius 10234, Lithuania

**Keywords:** interfacial water structure, hydrogen-bonded network, gold interface, electrochemistry, sum-frequency generation, atomic force microscopy, sputter deposition

## Abstract

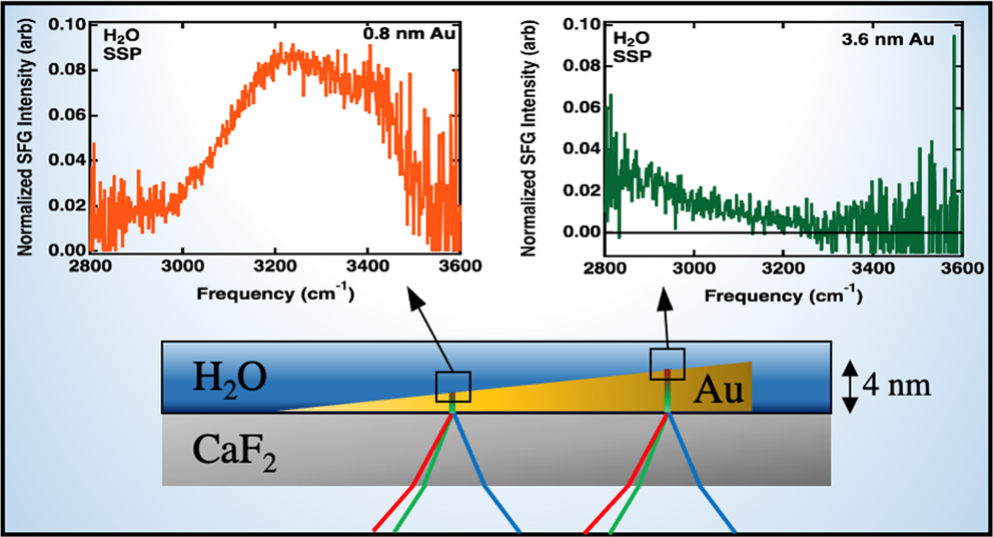

Water is an integral component in electrochemistry, in the generation of the electric double layer, and in the propagation of the interfacial electric fields into the solution; however, probing the molecular-level structure of interfacial water near functioning electrode surfaces remains challenging. Due to the surface-specificity, sum-frequency-generation (SFG) spectroscopy offers an opportunity to investigate the structure of water near working electrochemical interfaces but probing the hydrogen-bonded structure of water at this buried electrode–electrolyte interface was thought to be impossible. Propagating the laser beams through the solvent leads to a large attenuation of the infrared light due to the absorption of water, and interrogating the interface by sending the laser beams through the electrode normally obscures the SFG spectra due to the large nonlinear response of conduction band electrons. Here, we show that the latter limitation is removed when the gold layer is thin. To demonstrate this, we prepared Au gradient films on CaF_2_ with a thickness between 0 and 8 nm. SFG spectra of the Au gradient films in contact with H_2_O and D_2_O demonstrate that resonant water SFG spectra can be obtained using Au films with a thickness of ∼2 nm or less. The measured spectra are distinctively different from the frequency-dependent Fresnel factors of the interface, suggesting that the features we observe in the OH stretching region indeed do not arise from the nonresonant response of the Au films. With the newfound ability to probe interfacial solvent structure at electrode/aqueous interfaces, we hope to provide insights into more efficient electrolyte composition and electrode design.

## Introduction

Electrochemical processes are widely used in industries to drive chemical reactions, such as the transformation of abundant organic molecules to higher value products,^1^ to selectively enhance product ratios,^2^ and electroplating to produce resistant materials.^3^ Many electrode materials are utilized in electrochemistry, yet Au surfaces are attractive due to their high conductivity, corrosion resistance, and biocompatibility and because they are considered to be chemically inert.^4^ Gold is also more easily incorporated into biological systems than other noble metals,^5^ a trait which has led to its use in various pharmaceuticals.^6^ Gold nanoparticles and structured surfaces also contain plasmonic resonances, which can be tuned by changing the aspect ratio of the nanoparticle shape, or in the case of spherical nanoparticles by changing the diameter.^7^ Gold surfaces roughened via chemical etching can also greatly enhance the Raman and infrared (IR) response of adsorbed molecules, leading to surface-sensitive spectroscopic investigations of molecules.^8^ Many of these applications take place in aqueous media, where solvent orientation and surface interactions can affect the energetics of chemical reactions.^9^ Gold/aqueous interfaces play an important role in many industrial, pharmaceutical, and medical applications, but while the importance of water in driving electrochemical processes is widely recognized, the investigation of the fundamental water structure at electrochemical interfaces is hardly experimentally realized. Detailed molecular-level knowledge is needed to design and optimize electrolyte compositions and electrode structures to improve electrochemical processes.

Despite the many applications of gold surfaces, there are still open questions regarding the microscopic properties of the gold/water interface. The local hydrophilicity, for example, is still unknown for gold/water interfaces. While many studies have performed contact angle measurements for gold surfaces, these experiments measure the macroscopic wettability.^10^ Recent surface-specific spectroscopic measurements of geochemical surfaces show that minerals which display macroscopic hydrophilicity can contain microscopic hydrophobic patches, which influence the local solvent orientation and vibrational dynamics.^11^ This finding is corroborated by surface-specific investigations of self-assembled monolayers (SAMs) that show clear differences between the macroscopic and molecular-level properties, that is, that one cannot directly infer molecular-level information from macroscopic surface-tension experiments.^12^ Further information regarding the local water structure at gold surfaces is required to address microscopic surface properties of gold substrates, which can have macroscopic implications. The local adsorption/chemisorption of specific ions at H_2_O/metal interfaces has also been attributed to significantly alter the predicted double-layer capacitance from the established Gouy–Chapman (Stern) model, highlighting the need for a tool to study these environments.^13,14^ Surface-specific vibrational techniques, such as vibrational sum-frequency-generation (SFG), provide an opportunity to probe the orientation and hydrogen bonding strength of water in the interfacial region.^15,16^

While gold surfaces are employed in many technologies, surface-specific vibrational measurements of water at gold surfaces have remained elusive. The OH stretching mode of water, which contains resonances in the 3000–3700 cm^–1^ region, is a highly sensitive marker of the hydrogen-bonding strength.^16−18^ Accordingly, probing the OH stretch vibration of water at the gold/water interface would yield information regarding the local hydrogen bonding network at the surface; however, it has proven challenging to access this buried interface due to the optical properties of gold and water.^16,19^ In vSFG measurements, a visible and IR pulse are temporally and spatially overlapped at the interface, generating a third photon at the summed frequency.^20−24^ The air/water interface can easily be probed by having the visible and IR beams approach the interface from the air side.^25−31^ To probe a buried interface, the beams must propagate through one of the materials. The strong IR absorption of water results in complete loss of IR photons at penetration depths larger than a few μm in the hydrogen-bonded OH stretch spectral region (3000–3600 cm^–1^).^32^ While the non-hydrogen-bonded OH resonance or “free-OH” at the gold/water interfaces has been probed from the water side, the strong absorption of water has made it impossible to probe the hydrogen-bonded spectral region from the side.^32^ Alternatively, the gold/water interface can be probed through the substrate, which is common for vSFG of solid/liquid interfaces.^11,18,24,33−35^ However, this is complicated for gold surfaces due to the strong distortion of the interfacial electric fields as described by the Fresnel coefficients and the strong nonresonant SFG response of the highly polarizable conduction band electrons.^36^ For Au layers thicker than 5 nm, this leads to modulations of the SFG spectral response in the OH stretch region, which obscures the resonant OH stretch vibrations.^19^ Accordingly, it was thought impossible to probe the hydrogen-bonded network of water at the gold/water interface with SFG spectroscopy. The present study addresses this key issue and determines if it is possible and, if so, under which conditions can resonant OH vibrations at the gold/water interface be measured.

Previously vSFG has successfully been implemented to study sharp vibrational modes at the gold/water interface. These experiments have taken advantage of a technique developed by Dlott and co-workers,^37^ wherein the visible upconversion pulse is delayed by a few hundred fs with respect to the IR pulse. This greatly suppresses the nonresonant background resulting in near-background free vSFG spectra of the resonant vibrations.^19,37,38^ As such, Stark-active SAMs in the CN stretching region on gold substrates,^38−40^ which can report the local electrostatic potential, have been studied.^40^ Other monolayers have also been formed on gold surfaces and probed using vSFG, focusing on the CH,^38,41,42^ CD,^38^ and NO_2_^38^ functional groups. However, this approach is not possible for studying the broad resonances of hydrogen-bonded water. In a multiplex SFG experiment, a broadband IR pulse excites a series of vibrational modes that undergo free-induction decay (FID), decaying with the dephasing time.^43−45^ A narrowband visible pulse of picosecond duration then upconverts the FID in a Raman-like process resulting in the emission at the SFG wavelength.^46,47^ In addition to the FID of the resonant modes, an instantaneous nonresonant response is generated during the pulse overlap of the IR and visible pulses. By inserting a time delay between the IR and visible pulses, the nonresonant background is suppressed while the longer lived FID of narrow vibrational modes (typically a few ps) is still upconverted.^37,38^ However, this trick is not possible when probing hydrogen-bonded water, which displays sub-100 fs dephasing times at mineral/liquid interfaces,^48^ and thus exhibits a FID lasting only slightly longer than the nonresonant response during pulse overlap.

**Figure 1:**
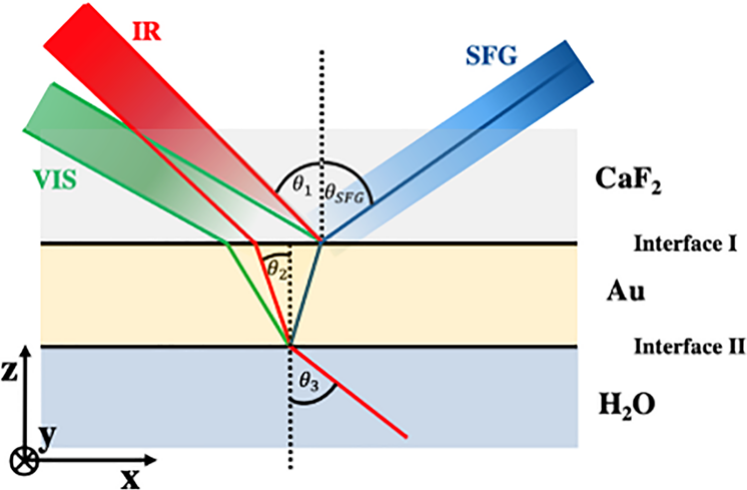
Schematic of the experimental geometry used for vSFG measurements at the CaF_2_/Au (varied thickness)/H_2_O interface.

A further complication is that the vSFG spectrum of a given interface is modulated by the Fresnel factors, which describe the local electric field enhancement at the interface. The Fresnel coefficients depend on the bulk linear refractive indices of each layer, which lead to a significant dispersion of the IR radiation in the OH stretching region and distort the nonresonant response.^19^ Calculating the Fresnel factors can help disentangle the nonresonant and resonant contributions to homodyned vSFG.^19,38,49^ The buried CaF_2_–gold–water interface constitutes a three-layer system comprising two interfaces, as shown in Figure 1. The full theoretical treatment of Fresnel factors for three-layer systems was described earlier by Backus et al. and calculated for a fixed gold thickness.^19^ They found that for the Al_2_O_3_/Au/H_2_O interface with a 5 nm gold film, the Fresnel factors obscured the resonant response of interfacial water.^19^ To quantify this effect in the present study, we calculated the frequency-dependent Fresnel factors for our system with both H_2_O and D_2_O as the bulk liquid and a varying gold film thickness. Our results differ from the work of Backus et al. due to the lower refractive index of CaF_2_ compared to that of Al_2_O_3_. Using the calculated Fresnel factors as a reference when interpreting the vSFG spectra is especially important when probing spectrally broad resonances such as interfacial water compared to narrow features, as the amplitudes of the Fresnel coefficients can be modulated on the frequency axis similarly to bulk water vibrational features.^19,50,51^ The Fresnel factors calculated for the present SFG experiment of the CaF_2_/Au/H_2_O interface are presented later.

SFG measurements were performed on thin gold films ranging in thickness from 0.4 to 8 nm at the CaF_2_/Au/H_2_O interface (Figure 1). The Au thickness gradient on the CaF_2_ substrate allowed us to systematically investigate at which Au thickness the vSFG resonant response of interfacial water molecules could be observed at the Ca/Au/H_2_O interface. The previous studies showed that the Fresnel factors, which describe the local electric field enhancement at the interface, dominate the spectra for the Al_2_O_3_/Au/H_2_O interface with a gold thickness of 5 nm.^19^ We observe similar effects for the thicker Au films but see the appearance of interfacial water resonances for thinner (∼<2 nm Au) Au films. To validate that these are resonant OH features, we also measured vSFG spectra of the gold films in contact with D_2_O (i.e., the CaF_2_/Au/D_2_O interface), which shows no resonances in the OH stretching region and calculated the frequency-dependent Fresnel factors for the interface, which can distort the vSFG spectra. Accordingly, we report the first measurement of resonant vSFG features in the hydrogen-bonded OH region of the Au/H_2_O interface, which will pave the way for future surface-specific studies of these frequently implemented gold/aqueous interfaces.

## Methods and Experimental Section

### Sample Cell and Sample Cleaning

Dry samples consisted of 50.8 mm diameter, 2 mm thick CaF_2_ windows (Knight Optical WCF5102) coated with a thickness-gradient Au film, mounted on 50.8 mm adjustable rotation mounts. The deposition process is described below. To measure the spectra of samples in contact with water, a 50.8 mm Teflon backplate (Thorlabs LAT500) was used in combination with polytetrafluoroethylene O-rings (APSO parts) and a 50.8 mm diameter, 2 mm thick CaF_2_ window with a Au gradient to form a liquid cell. Two holes drilled into the backplate allowed for the sample chamber to be filled or drained via Teflon tubing.

All glassware used in sample preparation and other sample components were cleaned in Nochromix solutions for a minimum of 30 min. Nochromix solutions were prepared by dissolving Nochromix cleaning reagent (Godax Laboratories) in concentrated sulfuric acid (Fisher Scientific, certified ACS plus grade). All cleaned glassware and sample cell components with the exclusion of gold-coated CaF_2_ were then rinsed with copious amounts of ultrapure water (18.2 MΩ per cm resistivity at 25 °C, 5 ppb total organic carbon) filtered with a Millipore system. To ensure that no residual acid was present, drops of water from the parts and glassware were tested using pH strips. Sample components were then dried using ultrapure N_2_ gas. All components, including the gold-coated CaF_2_, were then cleaned using an ozone generator (Ossila UV ozone cleaner) for 15 min to remove atmospheric organic contaminants.

### Laser Setup

The vSFG spectrometer used in this work to probe the CaF_2_/Au/H_2_O interface has been described in detail.^12,24^ In short, a Ti:sapphire oscillator (Coherent Micra-5) seeds a Ti:sapphire regenerative amplifier (Coherent Legend Elite Duo) which generates 25 fs, 800 nm, 6 mJ pulses at a 1 kHz repetition rate. Spectral resolution is achieved by narrowing the visible 800 nm pulses using a Fabry–Pérot etalon (TecOptics, Inc.). Broadband IR pulses are created using an optical parametric amplifier (Coherent OPeraA Solo), which is pumped by 3 mJ of the 800 nm beamline generated in the regenerative amplifier. The resulting IR pulses have a full-width-half-maximum bandwidth of ∼250 cm^–1^. To cover the OH stretching region (3000–3700 cm^–1^), multiple optical parametric amplifier (OPA) signal positions were used. To avoid burning the thin Au film, low pulse energies were used: at the sample, the visible pulse (centered at 792.5 nm) contained ∼2 μJ and the tunable IR pulses had pulse energies of ∼1 μJ. The spot size of the IR beam was ∼450 μm at the interface, with the visible beam slightly larger to ensure that the entire IR pulse area can be upconverted. The incident angles of the IR and upconversion beams at the Au/H_2_O interface (θ_2_ in Figure 1) were ∼40 and ∼35°, respectively. The vSFG responses were focused onto the slit of a spectrometer (Princeton Instrument, Acton SP2500) using a diffraction grating (600 grooves/mm, blazed at 500 nm) and imaged with a liquid nitrogen-cooled CCD camera (Princeton Instruments, model 7509-0001, 1340 × 400 pixels). The IR pulse profile was characterized using thick gold films (∼100 nm) or ZnO and used to normalize the raw vSFG data.

### Deposition of Thickness Gradient Au Films

**Figure 2:**
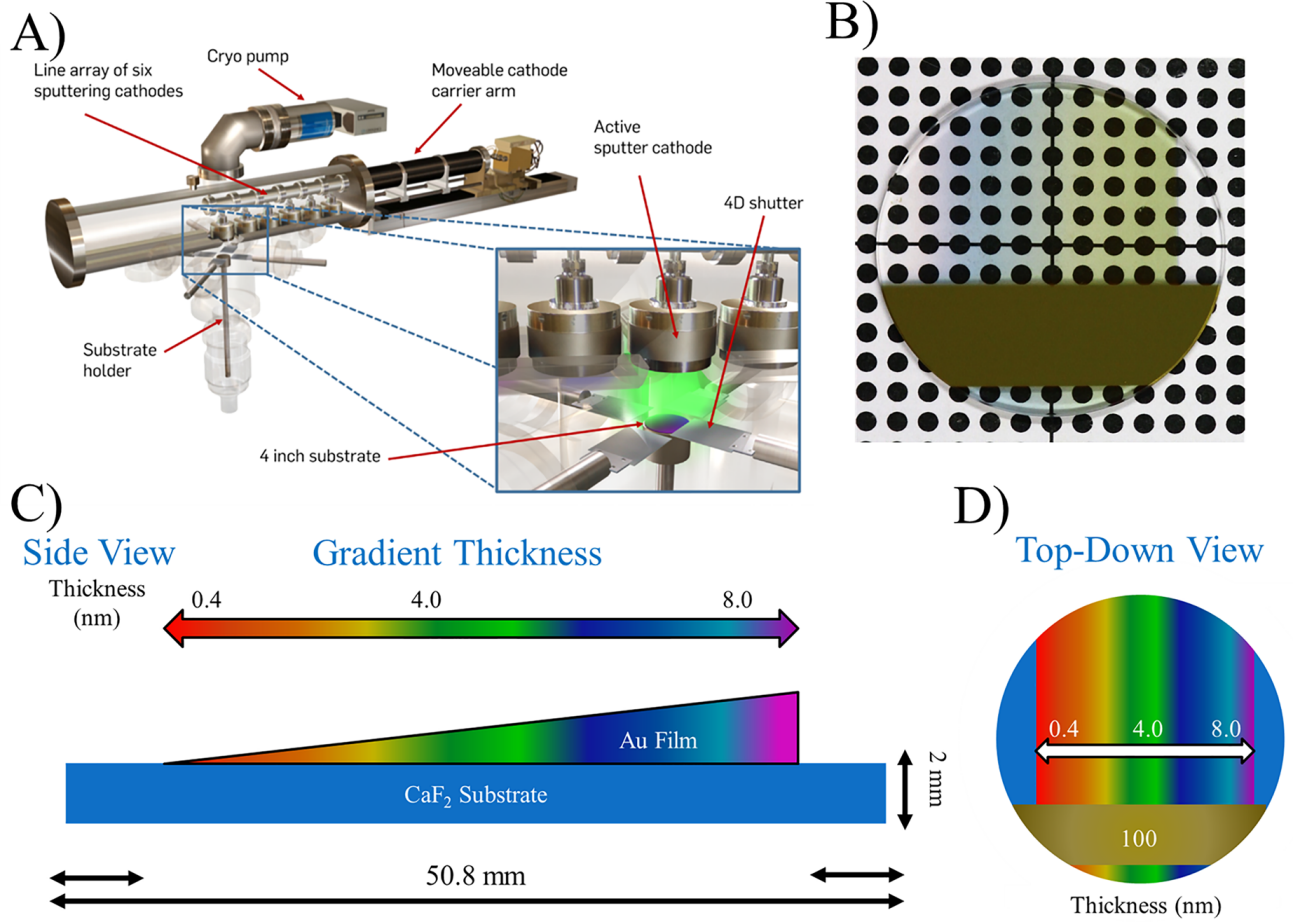
Schematic of the sputter system used for the deposition of Au thickness gradient films (“wedges”) and optical image of the gradient gold film on CaF_2_ named sample K1-4. (A) Magnetron sputtering setup used to create the gradient gold films: the automated shutters allow for a continuous thickness gradient film to be deposited. (B) Optical image of the thickness gradient gold film on CaF_2_ laid on a patterned display to help the eye detect the color changes. Thicker regions of the gradient film appear more yellow/gold, while thinner regions appear blue/purple. The 100 nm thick Au region used as a vSFG reference is observable at the bottom of the substrate as a horizontal strip. (C,D) Illustrate schematically the nominal Au thickness gradient in cross section and top view.

The Au thin films with thickness gradients were fabricated by magnetron sputtering from 101.6 mm diameter Au targets on 50.8 mm diameter, 2 mm thick CaF_2_ substrates. The deposition chamber base pressure was 2 × 10^–6^ Pa, the Ar deposition pressure was 0.66 Pam and the Ar (99.9999% purity) flow rate during the deposition was 60 standard cubic centimeters per minute (SCCM). The deposition was performed at room temperature. Direct current sputtering powers of 33 and 66 W were used to deposit two Au gradients with different maximum thickness. These powers resulted in a sputter rate of ∼0.1 and ∼0.2 nm/s, respectively (Figure 2). The wedge-type layers were made using a moving shutter which was set to shield the substrate and was then retracted (speed 2 mm/s) during deposition. This leads to the formation of a single wedge-type layer with a nominal thickness gradient spanning from 0 to 4 or 0 to 8 nm depending on the deposition rate, respectively. To generate a vSFG reference in the optical plane, an area of Au with a thickness of ∼100 nm Au was deposited on the lower part of the substrates. Two replica samples of each thickness were made: K1-1 and K1-2 were produced with films that vary in thickness from 0 to 4 nm and samples K1-3 and K1-4 span from 0 to 8 nm.

### AFM Experimental Section

AFM mappings were conducted under ambient conditions on a Bruker Bioscope, utilizing the Peak Force Tapping Mode at a 2 kHz resonant frequency with a ScanAsyst Air cantilever at 150 nm peak amplitude, a set point of 1.4 nN, and a scan rate of 0.5 Hz. The pristine gradient Au film was measured over three diverse regions of maximum 10 × 10 μm^2^ each, corresponding to three different layer thicknesses of 1.6, 2.0, and 2.8 nm for sample K1-2 and 3.2, 4.0, and 5.6 nm for sample K1-3.

### UV–Vis Experimental Section

UV–vis spectra were taken using a Hewlett Packard 8453 UV–vis spectrometer in transmission geometry. A home-built setup using a portable lamp source and a USB spectrometer was used to take reflection and transmission spectra of the gold films on CaF_2_.

### FTIR-Experimental Section

Optical characterization of the gradient films in the IR was accomplished using a Thermo Scientific Nicolet 8700 FT-IR spectrometer. Spectra were acquired by averaging five spectra with ∼4 cm^–1^ resolution in transmission geometry.

### Fresnel Factor Calculations

The Fresnel factors, which describe the electric field enhancements in the interfacial region, can significantly modulate the observed vSFG response, especially when probing metal surfaces.^19^ Calculating the frequency-dependent Fresnel factors can approximate enhancements in the nonresonant contribution to the total vSFG response, which can mimic features and/or obscure peaks in the vSFG spectrum.^19,38,49,52−54^ Comparing the frequency-dependent Fresnel factors with the collected vSFG spectrum can help separate resonant and nonresonant contributions to the total vSFG response.

The intensity of the vSFG response (*I*_SFG_) is proportional to the intensities of the incident visible (*I*_vis_) and IR beams (*I*_IR_), as well as the square of the second-order nonlinear susceptibility, χ_*ijk*_^(2)^, which is modulated by the local field effects, as described by the Fresnel factors *L*_ii_ (eq 1)
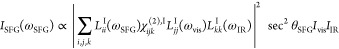
1here, the superscripts I and II denote the CaF_2_/Au and Au/H_2_O interfaces, respectively (Figure 1). *i*, *j*, and *k* are the coordinates of the reference frame which translate to the laboratory *x*, *y*, and *z* coordinates shown in Figure 1. In vSFG measurements, the polarization of the visible and IR beams can be rotated to probe different elements of the second-order nonlinear susceptibility tensor, χ_*ijk*_^(2)^.^55^ For achiral interfaces, 7 of the possible 27 elements of the χ_*ijk*_^(2)^ tensor are non-zero and 4 are unique. In our experiments, we measured SSP and PPP experimental spectra, where the light polarizations are listed in order of decreasing photon energies. SSP and PPP spectral intensities are given by eqs 2 and 3
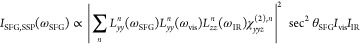
2
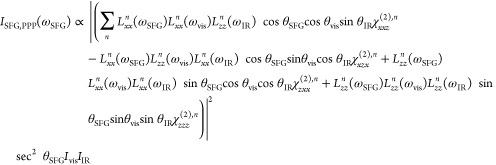
3where *n* denotes interface I or II, θ_IR_ and θ_vis_ are the incident angles of the IR and visible beams with respect to the surface normal, and θ_SFG_ is the calculated angle of the emitted SFG beam (Figure 1). *L*_*xx*_^I^, *L*_*yy*_^I^, and *L*_*zz*_^I^ are the frequency-dependent Fresnel coefficients for interface I described by eqs 4–6
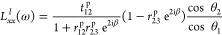
4
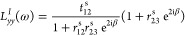
5
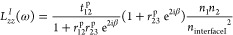
6Here, ω is the photon frequency, *r*_*ij*_^p^, *r*_*ij*_^s^,*t*_*ij*_^p^, *t*_*ij*_^s^ are the linear reflection and transmission coefficients between media *I* and *j*, *n*_1_ and *n*_2_ are the complex refractive indices of CaF_2_ and Au (Figure 1), and *n*_interfaceI_ is the refractive index of the interfacial layer. The optimal choice of the interfacial refractive index is still being debated. Here, we chose to use the average of both media between materials 1 and 2. We note that other authors have chosen to use the higher value refractive index,^19^ defined their own approximation,^55,56^ or have not stated their choice of definition for the interfacial refractive index.^49,52−54^ The β term is a phase difference factor, defined by eq 7
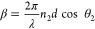
7here, λ is the photon wavelength, *d* is the thickness of the gold film, and θ_2_ is shown in Figure 1. The Fresnel coefficients for the second interface are defined by eqs 8–10^19^
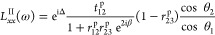
8
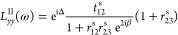
9
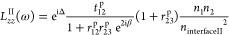
10

The addition of the e^iΔ^ term accounts for the phase difference between the vSFG response generated at interfaces I and II and is wavelength dependent (eqs 11–13).^19^ Due to the wavelength dependence, there are separate definitions for the SFG, visible, and IR photons
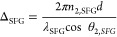
11
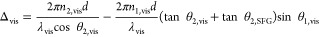
12
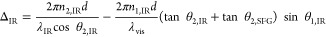
13

Using this formalism, we generated the individual Fresnel coefficients and total Fresnel factors for the CaF_2_/Au/H_2_O interface using PPP and SSP polarization combinations. The intermediate Au film thickness was varied from 0.4 to 100 nm in these calculations.

## Results and Discussion

**Figure 3:**
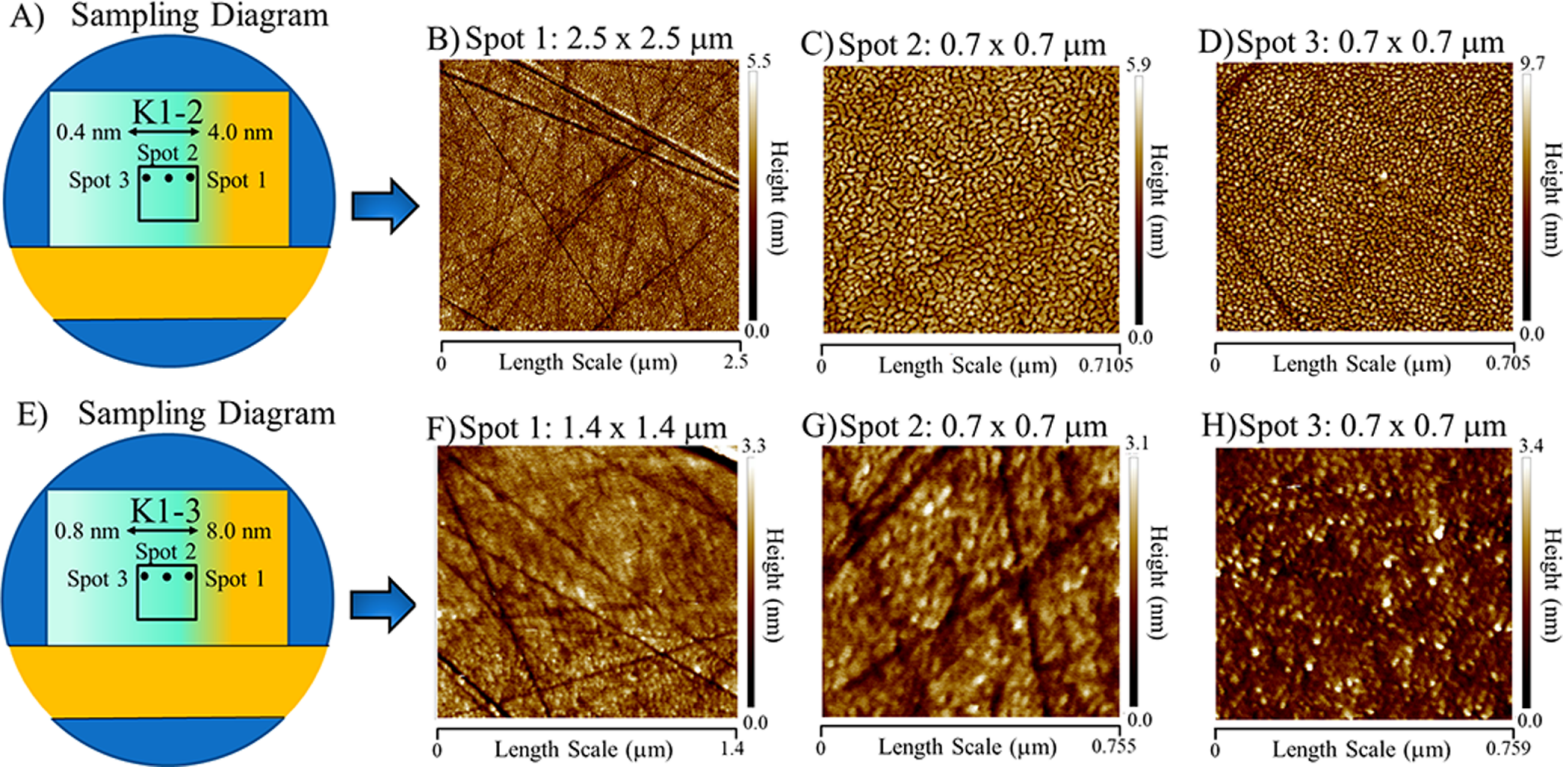
Sampling diagram showing the regions of both samples which were imaged using AFM and the resulting images. (A,E) show the regions sampled with AFM, (B–D) are the resulting AFM images from spots 1–3 for sample K1-2, and (F–H) are the AFM images for spots 1–3 for the thicker K1-3 sample.

Relatively thick Au film can be made with high surface quality and crystallinity. However, such films have high IR reflectivity and a large nonresonant contribution to the vSFG spectra from conduction band electrons. These decrease as the Au thickness decreases but so does the structural integrity and homogeneity of the film. The minimum thickness needed to form a uniform gold film with a (111) termination is 5 nm. But at this thickness, the vSFG response is dominated by the nonresonant contribution.^19^ The goal of the present investigation was to determine how thin the gold film needs to be in order to observe a resonant water response. The gradient film allows the gold thickness to be systematically varied by translating the sample across the vSFG beamline to determine at which nominal gold thickness the resonant OH vibrations of water can be successfully observed at the Au/H_2_O interface. Two sets of identical samples were fabricated, with gradients that ranged from 0 to 4 nm (K1-1 and K1-2) and 0 to 8 nm (K1-3 and K1-4). Each experiment was performed with a freshly prepared sample. As illustrated in Figure 3, the generated gold films were a total of 40 mm wide with an increasing thickness across the gradient. Before performing the vSFG experiments on the CaF_2_/Au/H_2_O system, we characterized the gradient gold films with linear spectroscopic and microscopic methods. The film homogeneity and roughness were characterized with AFM measurements utilizing a peak force tapping method. Due to the large diameter of our samples, only the middle region of the gradient could be sampled using our AFM instrument (Figure 3A).

We performed the AFM measurements on three different regions of each of the samples, as illustrated in Figure 3, corresponding to approximate Au thicknesses of 1.6, 2.0, and 2.4 nm for sample K1-2 and 3.2, 4.0, and 4.8 nm for sample K1-3. Although we could not image the entire sample, the regions probed provide insight into the film morphology for the important range of film thicknesses (∼1.6 to 5.6 nm). For the thicker depositions (Figure 3B,F–H), we found a smooth (<∼3 nm height fluctuations across the area sampled including polishing grooves of the substrate), continuous film, with grooves on the CaF_2_ substrate left from chemical polishing. These are highly uniform regions, and the most obvious morphological features arise from the substrate itself. Spot 3 for the thicker K1-3 sample (∼3.2 nm) begins to show a rougher surface morphology. For the thinner regions on sample K1-2 (Figure 3C,D), a clear change occurs, with the appearance of a popcorn-like surface layer and accompanying larger local variations in height. From AFM images, the films appear continuous in nature on the down to ∼1.6 nm, although they do not exhibit a pristine crystalline (111) surface. The popcorn-like structures were characterized further by imaging a few regions of Figure 3D at higher resolution, as shown in Figure S1. These images showed that the popcorn structure is composed of hemispheres terminating the top-most layer with a diameter of ∼11 nm and height of ∼4 nm. We note that the local height variations of the CaF_2_ substrate are on the order of a few nm, which contribute to the large height changes between individual particles as well (Figure S1). The visual changes in the film itself (Figure 2B) confirm that we were able to deposit a gradient of gold across the sample smoothly varying from 0 to 4 or 8 nm at the end of the 40 mm length scale associated with the deposition. AFM imaging shows that uniform gold films on CaF_2_ substrates down to at least 1.6 nm can be made that transition from a uniform film to a more structured roughened film at around 3 nm film thickness.

**Figure 4:**
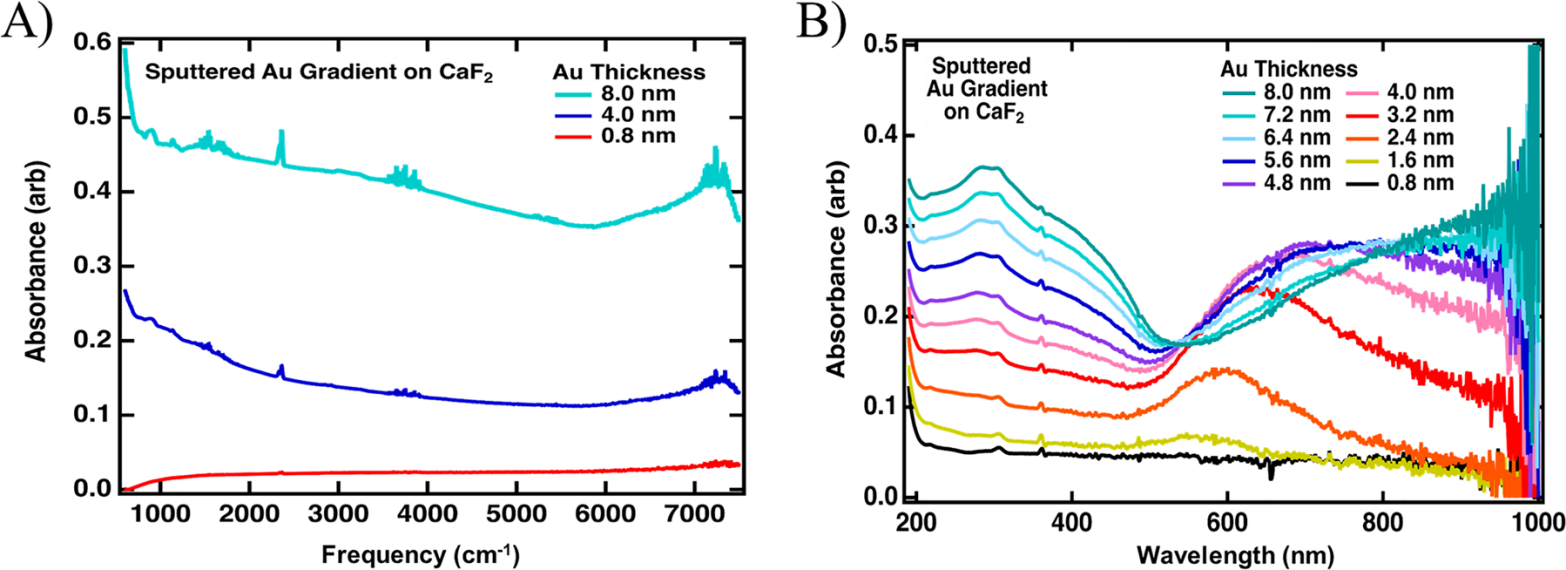
Optical properties for a range of gold thicknesses in the IR and visible frequency windows at normal incidence. (A) FTIR absorption measurements show relatively flat spectra in the IR, with very little (∼0.02%) to almost half of the IR photons being lost for thin (0.8 nm) and thick (8.0 nm) regions of the film, respectively. (B) UV–vis spectra of the gold films. For thicker depositions, two prominent peaks are observed at ∼300 and 700 nm, while thinner regions show a broad absorption peak, which blue shifts as the film thickness decreases from ∼630 to 590 nm.

An important consideration for the nonlinear spectroscopic measurements is the optical properties of the gold films. Accessing the gold/water interface through the substrate (Figure 1) involves transmittance of the IR, visible, and vSFG photons through the deposited gold layer, requiring knowledge of the optical properties before performing vSFG measurements. FTIR absorption measurements show that the 8.0 nm film results in a ∼65% loss of IR photons in the OH stretching region, while the 0.8 nm film absorbs/reflects ∼2% of the incoming IR beams (Figure 4A). The relatively featureless nature of the FTIR absorption spectra also suggests that IR absorption by the gold layer would not affect the spectra shape of our IR pulses before vSFG generation. To investigate the absorption of the vSFG and visible (792.5 nm) photons, we acquired UV–vis spectra for 8.0 to 0.8 nm thick gold films at normal incidence (Figure 4B). We also acquired UV–vis reflectance measurements at a range of angles of incidence, all of which displayed relatively flat behavior in the vSFG photon wavelength window from ∼620 to 640 nm (Figure S2). For films with thicknesses from 8.0 to 4.0 nm, a well-resolved absorption feature was present at ∼320 nm with two distinct local maxima. As the film thickness decreased, only the narrow red edge peak remained. At ∼4.0 nm, the ∼320 nm absorption feature faded, and a broad absorption peak emerged as the dominant spectral feature which shifted in central wavelength from ∼630 to 600 nm as the gold film approached 1.6 nm. At 0.8 nm, the spectrum was almost featureless. We hypothesize that the broad peaks at ∼630 to 600 nm for 3.2 to 1.6 nm films indicate plasmonic behavior, as has been suggested recently by Baker et al. for similar sputtered gold films.^38^ This also correlates with the change in film morphology seen from our AFM measurements (Figure 3). Here, the film morphology transitioned from smooth gold surfaces at thicker thicknesses to more rough surfaces below ∼3 nm, with gold hemispheres populating the surface. VSFG photons in our experiments were generated from ∼620 to ∼640 nm, which spectrally overlap with the observed plasmon resonance. This allowed for plasmonic enhancement of the generated vSFG response, which is discussed in more detail below.

**Figure 5:**
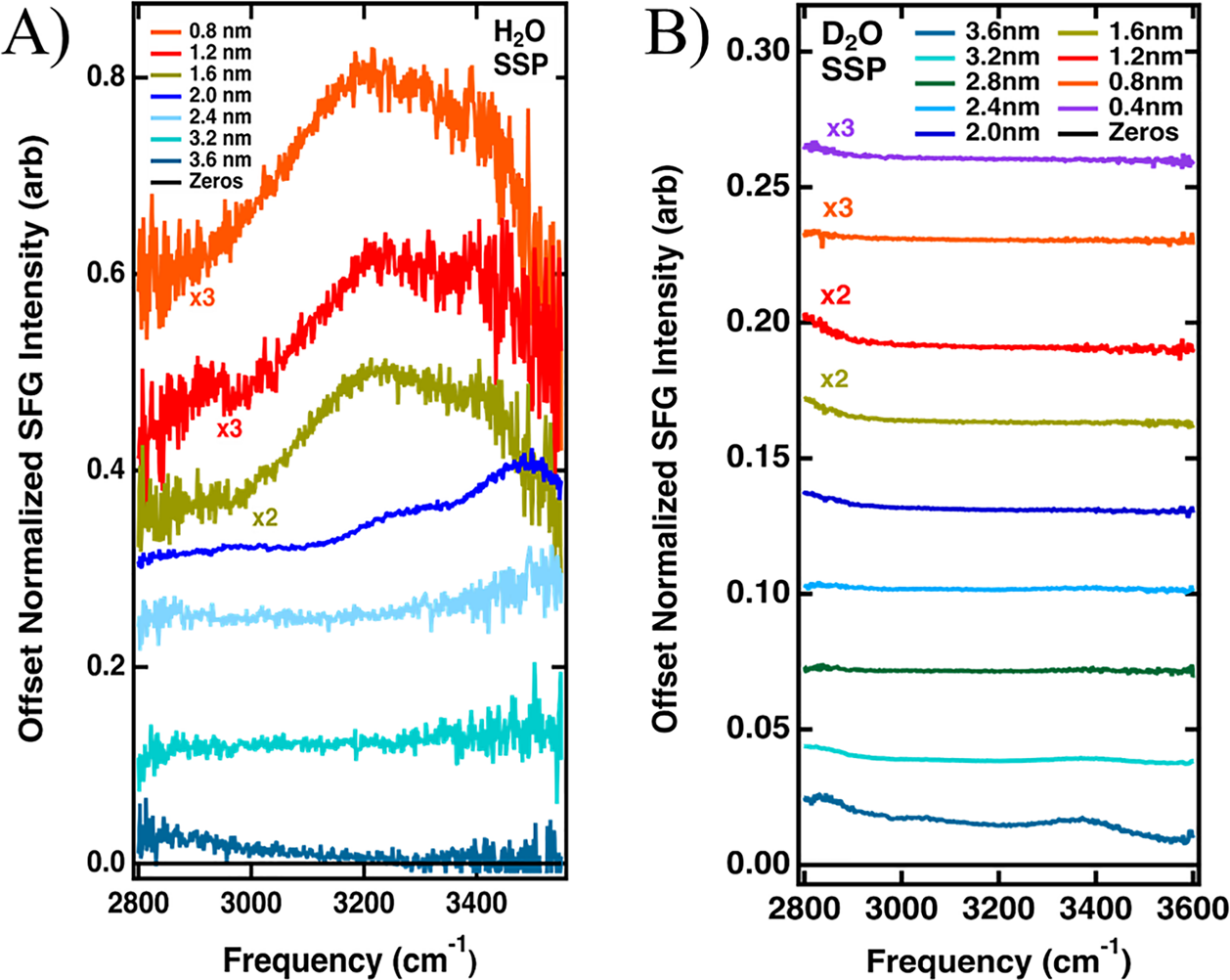
vSFG measurements of the (A) CaF_2_/Au/H_2_O and (B) CaF_2_/Au/D_2_O interfaces at various gold thicknesses taken using the SSP polarization combination. At the CaF_2_/Au/H_2_O interface, thicker films display no response, and at ∼1.6 nm Au thickness, interfacial H_2_O becomes detectable. The CaF_2_/Au/D_2_O interface shows a flat vSFG response, except for the 3.6 nm film.

To probe the interfacial water resonances across the gradient gold films, we performed vSFG measurements on the OH stretching region. We chose to use the SSP polarization combination since the nonresonant response from Au is much weaker in SSP than in PPP due to the node in the electric field present at the surface for noble metals for S polarized light. Thus, while the PPP polarization is dominated by the strong Au response, the SSP polarization combination allows for easier detection of resonant OH stretching vibrations.^57^ To highlight the appearance of resonant OH stretches over the nonresonant background, we compare the IR profile-normalized vSFG spectra (non-Fresnel factor corrected) measured for CaF_2_/Au/H_2_O and CaF_2_/Au/D_2_O interfaces in Figure 5. CaF_2_/Au/H_2_O and CaF_2_/Au/D_2_O exhibit similar refractive indexes, making this a better comparison than to the CaF_2_/Au/air interface.^19^ As in the previous study,^19^ we were not able to detect a surface water response for gold film thicknesses of >5 nm. For this reason, we focus on the 3.6 nm and thinner regions of the sample here. For the CaF_2_/Au/H_2_O interface, we found a low-amplitude flat nonresonant response for gold films of 3.6 to 2.4 nm (Figure 5A). At 2.0 nm Au, two broad features began to appear in addition to the nonresonant response of Au. At film thicknesses of 1.6 nm and below, we observed the familiar OH stretch resonances associated with interfacial water response with a main peak centered at ∼3200 and ∼3400 cm^–1^. The fact that the amplitude of the ∼3200 cm^–1^ peak, which is associated with more strongly hydrogen-bonded surface water, is greater than the ∼3400 cm^–1^ weakly hydrogen-bonded feature suggests that interfacial water at the gold interface does not experience a substantial decay of the hydrogen-bonding network strength.^16−18^ The spectral shape remains constant under ∼1.6 nm. While the OH stretch is vibrationally resonant in the ∼3000–3700 cm^–1^ frequency domain, the heavier OD stretch is significantly red-shifted and vibrates in the ∼2000–2700 cm^–1^ window.^32^ By probing the CaF_2_/Au/D_2_O interface with IR pulses in the OH stretching region, any vSFG response from the solvent can be removed. To experimentally verify that the response in the OH stretching region seen in Figure 5A originates from surface waters, we sampled the CaF_2_/Au/D_2_O interface (Figure 5B) with the IR pulses of the same frequency. For D_2_O, film thicknesses from 0.4 to 2.8 nm resulted in a flat nonresonant response that increased in intensity with film thickness. At 3.2 nm, a slight hump at ∼3400 cm^–1^ began to emerge, and at 3.6 nm, the intensity of this feature increased. The location of this feature and maximum amplitude in the D_2_O spectra was not as pronounced but similar to what previous authors have observed for 5 nm Au films deposited on Al_2_O_3_.^19^ Although the CaF_2_/Au/D_2_O interface was not featureless for the thicker regions (3.6 to 3.2 nm), it was flat in the 1.6 and thinner regions of the sample, where the interfacial H_2_O spectra could be collected.

**Figure 6:**
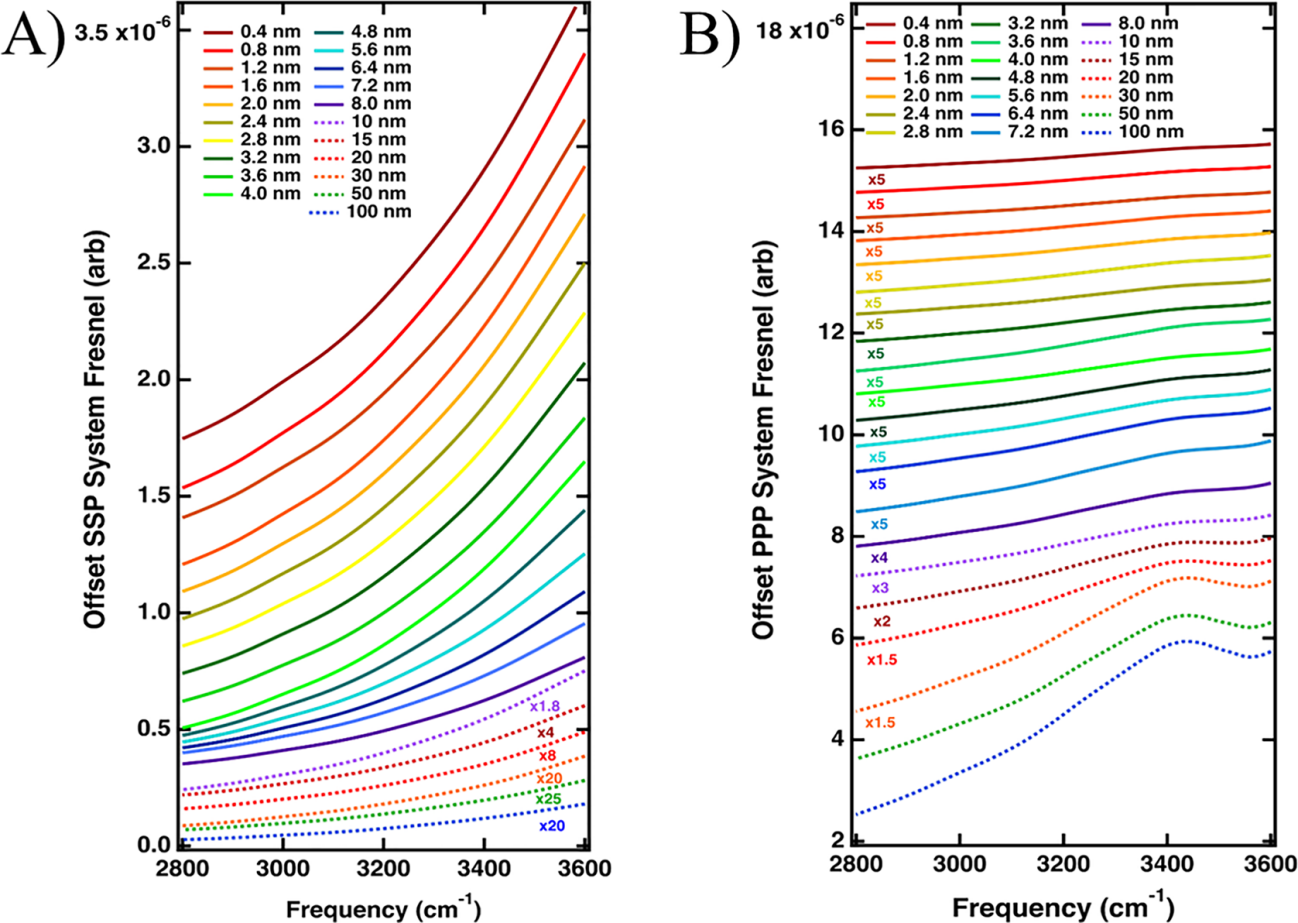
Total frequency-dependent Fresnel factors simulated for the CaF_2_/Au/H_2_O interface for Au film thicknesses from 0.4 to 100 nm selecting (A) SSP and (B) PPP experimental geometries. The largest Fresnel amplitudes are seen for thin Au films in SSP polarization (A), especially on the blue side of the frequency window. PPP Fresnel factors show a feature similar to weakly hydrogen-bonded water for thick Au films (100–8 nm) yet are weaker in amplitude across the OH bonding region than SSP Fresnel factors.

To fully rule out that the observed spectral features could be due to the Fresnel coefficients and not resonant water features, we calculated the frequency-dependent Fresnel factors for the CaF_2_/Au/H_2_O interface. We followed the same methodology as previous authors who have simulated Fresnel coefficients for a model three-layer system.^19,58^ The external incident angles (θ_1_ in Figure 1) of ∼55 and 65° are used for the IR and visible beams, respectively. These result in internal incident angles (θ_2_ in Figure 1) of 40 and 35° calculated using Snell’s law for the IR and visible beams, respectively. Beam angles of incidence were fixed at the same values as used in our experiment, allowing for the frequency-dependent Fresnel factors to be calculated in the OH stretching region (Figure 6). The complex refractive indices for Au^59^ and H_2_O/D_2_O^32^ were incorporated into the calculations. The interfacial refractive index, which is required in these calculations and has remained controversial due to the inability to experimentally measure this quantity, was taken as the average complex refractive index of the two media which form interfaces I and II in Figure 1. The Fresnel factors for the individual interfaces (Figure S3) can be found in the Supporting Information. Our simulations show that the total Fresnel factors of the system (interface I + II) are quite small and are approximately four times greater in amplitude across the OH stretching region for vSFG measurements using PPP geometry. This corresponds to the larger enhancement from the gold primarily in PPP geometry. Interestingly, the largest enhancement of the interfacial electric fields can be found for the thinnest Au coatings in SSP polarization and for the thickest Au films when the PPP geometry is simulated. For the SSP Fresnel factors, the enhancement factors retain a similar shape through all Au film thicknesses investigated (0.4 to 100 nm), with a rising edge on the blue side of the spectrum. Dividing the normalized vSFG by these curves would slightly reduce the vSFG spectrum on the ∼3400 cm^–1^ edge (Figure S5). The PPP Fresnel factors, however, do contain a significant spectral shape and as the Au film thickness is increased a feature appears at ∼3400 cm^–1^ which becomes well defined above film thicknesses of ∼20 nm. This leading edge on the blue side of the OH spectrum and peak at ∼3400 cm^–1^ was also observed by Backus et al. at the Al_2_O_3_/Au/H_2_O interface in PPP geometry.^19^ This could explain why the resonant features of interfacial water appear in SSP and not in the PPP polarization combination. We stress that the Fresnel factors represent the interfacial electric field enhancements, which can augment the produced vSFG spectra from the surface; however, they do not predict the second-order nonlinear susceptibility, χ^(2)^. For this reason, the calculation of the Fresnel factors from the interface suggests again that we are indeed able to sample interfacial water at the gold interface using vSFG for thin gold films.

**Figure 7:**
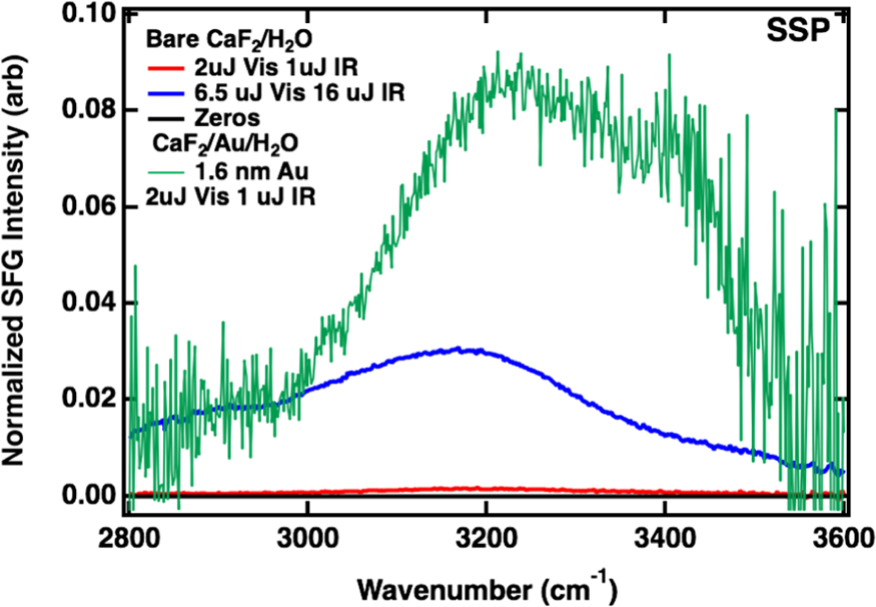
vSFG spectra of the CaF_2_/Au/H_2_O and CaF_2_/H_2_O interfaces using standard experimental pulse energies and highly attenuated visible and IR pulses. All traces are plotted on the same scale for amplitude comparison.

The ability to capture vSFG spectra at the Au/H_2_O interface using such low IR visible pulse energies (1 and 2 μJ) is remarkable. To understand the origin of this effect, we also studied the bare CaF_2_/H_2_O interface for comparison (Figure 7). We probed the CaF_2_/H_2_O interface using our standard IR and visible pulse energies (∼16 and 6.5 μJ, respectively) and the same conditions as were used for the gradient gold samples. If the second-order nonlinear susceptibility, χ^(2)^, remains constant, the intensity of the vSFG response should scale linearly with the intensity of the visible and IR beams.^20,60^ While the 1.6 nm Au CaF_2_/Au/H_2_O sample plotted in Figure 7 has 16 times less energetic IR pulses and ∼3 times less intense visible pulses, the overall signal strength is ∼4 times stronger than the bulk water spectra acquired using high pulse energies. This suggests that the vSFG response is amplified ∼200 times via plasmonic enhancement, enabling acquisition of vSFG spectra at such low pulse energies. For comparison, the CaF_2_/H_2_O spectrum acquired using the same low IR and visible pulse energies used in the CaF_2_/Au/H_2_O experiments is much weaker in intensity. While the vSFG spectrum measured for CaF_2_/H_2_O matches well with previous measurements in the literature,^61−63^ the spectral shape observed at the CaF_2_/Au/H_2_O interface is quite different, with more intensity in the ∼3400 cm^–1^ shoulder region, as discussed earlier. This further validates that this response results from water in contact with gold and not water in contact with CaF_2_ in between the gold islands on the sample.

## Conclusions

Contrary to previous findings, we have demonstrated that it is indeed possible to measure the resonant OH water response of hydrogen-bonded water in contact with gold. For this purpose, we fabricated gradient gold films sputtered on CaF_2_ with a thickness from 0 to 8 nm to determine which film thicknesses was appropriate for the vSFG measurements. These gradient films were characterized using AFM, where we found that the films were continuous and smooth at thicknesses above ∼3.6 nm and then transitioned to highly structured hemispheres at lower gold thickness depositions. This transition in the gold film morphology was accompanied with the growth of plasmonic activity, which we observed using UV–vis spectroscopy at normal angles of incidence. FTIR spectra indicate that the sample transparency in the IR is better than 40% at these thicknesses.

The vSFG measurements show that we were able, for the first time, to probe the hydrogen-bonded region of OH stretching vibrations of water at the buried gold/water interface for thin gold films. The result was confirmed using D_2_O, which shows a flat featureless response in the OH stretching region. Calculation of the frequency-dependent Fresnel factors further confirmed that the intensity in the OH stretching region does indeed originate from surface waters at the gold interface. Lastly, we correlate the ability to capture vSFG at low pulse energies with the plasmonic behavior of our thin gold films, which suggests a plasmonic enhancement factor of about 200. While it was previously thought to be impossible to probe hydrogen-bonded water at the buried gold/water interface, this study shows that this can indeed be done for thin <2 nm gold films. This finding opens a path for future studies to characterize the molecular structure of water in contact with the highly utilized gold surfaces.

## Supporting Information Available

(1) AFM-Deduced Hemisphere Dimensions. (2) UV–vis Measurements at vSFG Experimental Geometry. (3) Independent Fresnel Factors from Interface I and II . (4) Comparing SSP Fresnel Factor to Interfacial H_2_O Spectra (PDF)
