# Supplementary material for: Probing the Gold/Water Interface with Surface-Specific Spectroscopy
Source: ACS Phys Chem Au. 2023 Jan 4;3(1):119–29. doi: 10.1021/acsphyschemau.2c00044 (PMC9881240; doi:10.1021/acsphyschemau.2c00044)
Supplement: Supplementary file 1 — pg2c00044_si_001.pdf [file pg2c00044_si_001.pdf]

Probing the Gold/Water Interface with Surface Specific Spectroscopy

Stefan M. Piontek[1,5], Dennis Naujoks[2], Tadneem Tabassum,[1] Mark J. DelloStritto[3], Maximilian Jaugstetter[1], Pouya Hosseini[4], Manuel Corva[1], Alfred Ludwig[2], Kristina Tschulik[1], Michael L. Klein[3], Poul B. Petersen[1]*

[1]Faculty of Chemistry and Biochemistry, Ruhr-Universität Bochum, 44801 Bochum, Germany
[2]Faculty of Mechanical Engineering, Institute for Materials and ZGH, Ruhr-Universität Bochum, 44801 Bochum, Germany
[3]Institute for Computational Molecular Science, Temple University, Philadelphia, Pennsylvania, 19122, United States
[4]Max-Planck-Institut für Eisenforschung GmbH, 40237 Düsseldorf, Germany
[5] Light Conversion Inc., Vilnius, Vilnius City Municipality, 10234, Lithuania

Corresponding Author: poul.petersen@ruhr-uni-bochum.de

Table of Contents:

1. AFM Deduced Hemisphere Dimensions
2. UV-Vis Measurements at vSFG Experimental Geometry
3. Independent Fresnel Factors from Interface I and II
4. Comparing SSP Fresnel Factor to Interfacial H_2O Spectra

AFM Deduced Hemisphere Dimensions

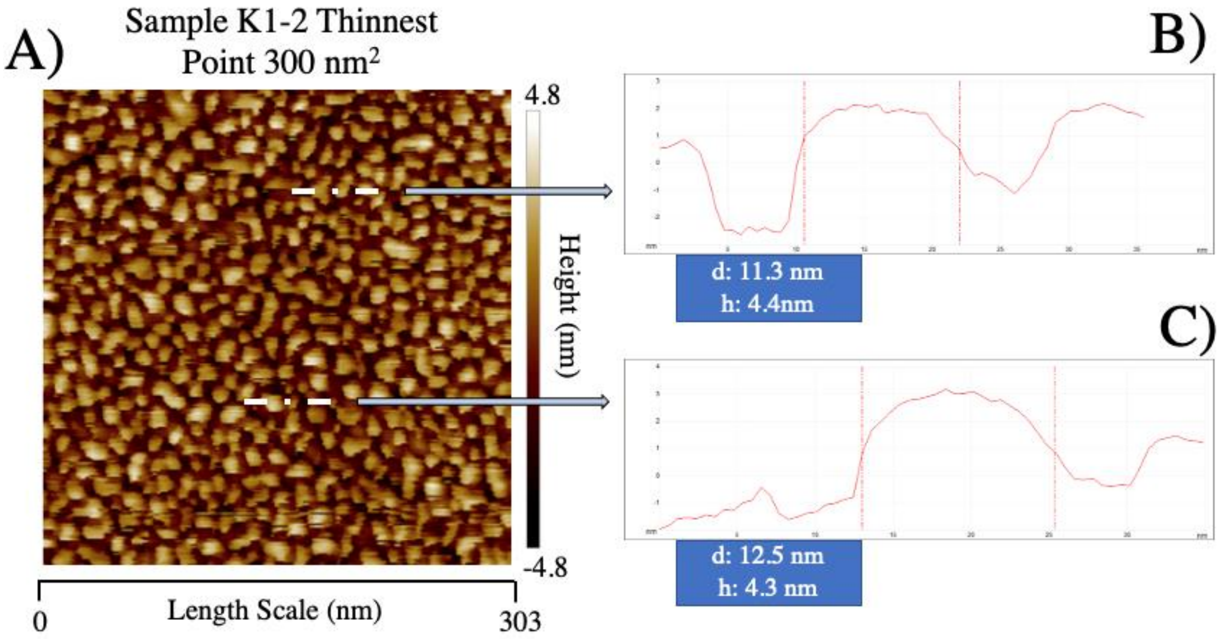

Figure S1:AFM image and corresponding height analysis of hemispheres found on the ~1.6/2.0 nm region of sample K1-2. Two hemispheres highlighted by a white dashed line and their corresponding height profiles are shown in graphs B) and C), respectively. From the height profiles hemisphere features can be estimated.

UV-Vis Measurements at vSFG Experimental Geometry

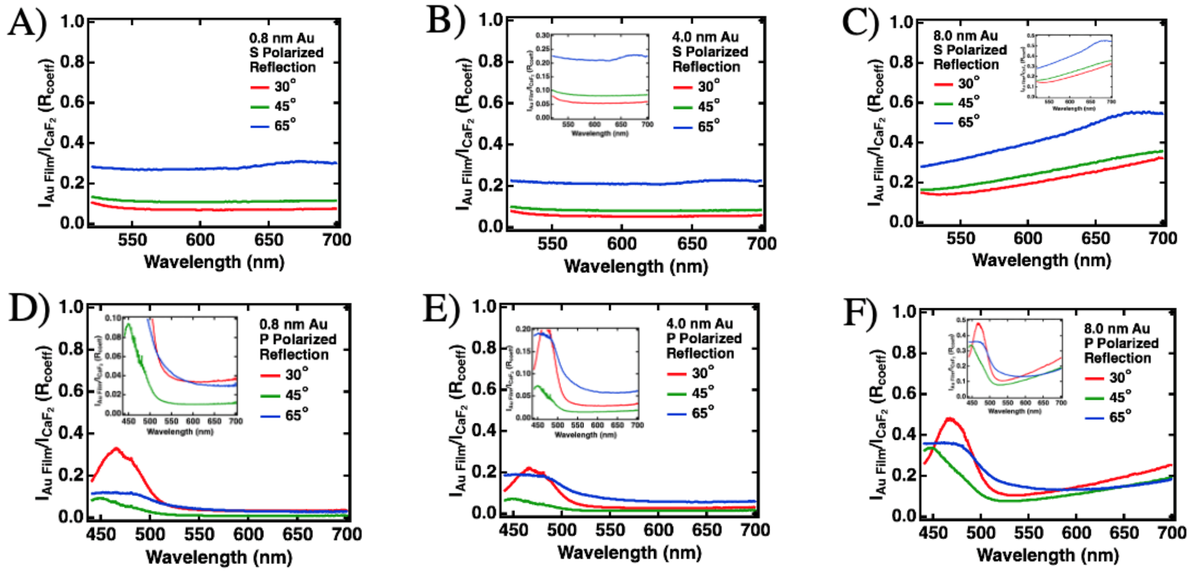

Figure S2: UV-Vis Reflectance measurements taken at a range of angles of incidence. Insets show the same graph with a reduced y-axis. **A-C)** Display a range of **A)** thin 0.8 nm, **B)** medium 4.0 nm, and **C)** thick 8.0 nm samples at angles of incidence of 30°, 45°, and 65° for S polarized light. **D-F)** Show the same measurements taken with P polarized light. All reflectance measurements show no significant spectral structuring in the 620-640 nm range where vSFG photons in our experiments are generated.

Independent Fresnel Factors from Interface I and II

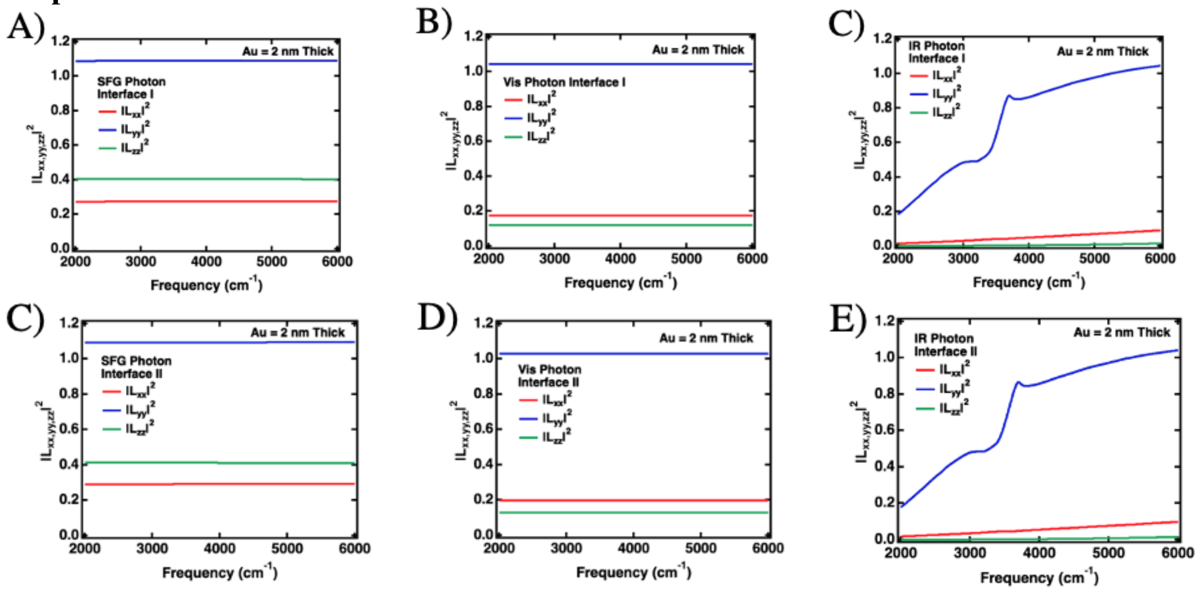

Figure S3: Generated frequency dependent Fresnel Factors for interfaces I (**A-C**) (CaF_2/H_2O) and II (**D-E**) (Au/H_2O) for each photon associated with the vSFG process. Flat curves for the visible and vSFG photons indicate the narrow band nature of the photons and the relative frequency independence of the optical constants associated with these materials in the visible.

Comparing SSP Fresnel Factor to Interfacial H_2O Spectra

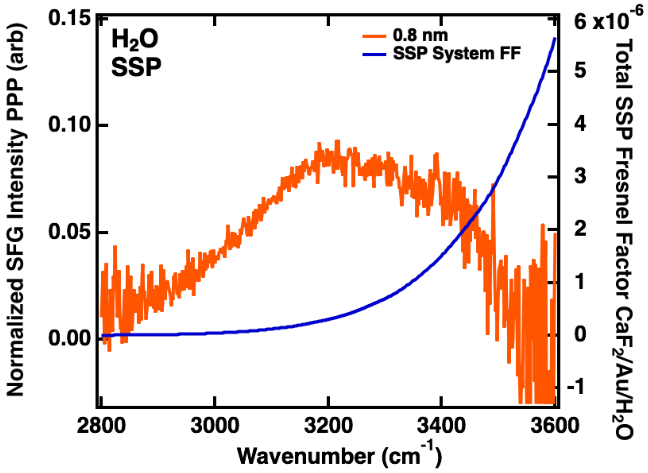

Figure S5: Comparison of the IR profile normalized SFG spectra from the 0.8 nm Au region of the gradient film and the corresponding frequency dependent Fresnel Factor. There is no obvious similarity in the normalized vSFG spectra from the $CaF_2/Au/H_2O$ interface.
